# Risk factors for peripheral venous catheter-related phlebitis stratified by body mass index in critically ill patients: A *post-hoc* analysis of the AMOR-VENUS study

**DOI:** 10.3389/fmed.2022.1037274

**Published:** 2022-11-28

**Authors:** Masahiro Kashiura, Hideto Yasuda, Takatoshi Oishi, Yuki Kishihara, Takashi Moriya, Yuki Kotani, Natsuki Kondo, Kosuke Sekine, Nobuaki Shime, Keita Morikane

**Affiliations:** ^1^Department of Emergency and Critical Care Medicine, Jichi Medical University Saitama Medical Center, Saitama, Japan; ^2^Division of Clinical Research Education and Training, Clinical and Translational Research Center, Keio University Hospital, Tokyo, Japan; ^3^Department of Intensive Care Medicine, Kameda Medical Center, Chiba, Japan; ^4^Department of Intensive Care Medicine, Chiba Emergency Medical Center, Chiba, Japan; ^5^Department of Medical Engineer, Kameda Medical Center, Chiba, Japan; ^6^Department of Emergency and Critical Care Medicine, Graduate School of Biomedical and Health Sciences, Hiroshima University, Hiroshima, Japan; ^7^Division of Clinical Laboratory and Infection Control, Yamagata University Hospital, Yamagata, Japan

**Keywords:** body mass index, catheter-related infections, catheters, intensive care units, phlebitis

## Abstract

**Introduction:**

Phlebitis is an important complication in patients with peripheral intravascular catheters (PIVCs). Although an association between body mass index (BMI) and phlebitis has been suggested, the risk of phlebitis according to BMI has not been well elucidated. Therefore, in this study, we analyzed the risk of phlebitis according to BMI in patients in the intensive care unit (ICU).

**Materials and methods:**

This study undertook a secondary analysis of the data from a prospective multicenter observational study assessing the epidemiology of phlebitis at 23 ICUs in Japan. Patients admitted into the ICU aged ≥18 years with a new PIVC inserted after ICU admission were consecutively enrolled and stratified into the following groups based on BMI: Underweight (BMI < 18.5 kg/m^2^), normal weight (18.5 ≤ BMI < 25.0 kg/m^2^), and overweight/obese (BMI ≥ 25.0 kg/m^2^). The primary outcome was phlebitis. The risk factors for phlebitis in each BMI-based group were investigated using a marginal Cox regression model. In addition, hazard ratios and 95% confidence intervals were calculated.

**Results:**

A total of 1,357 patients and 3,425 PIVCs were included in the analysis. The mean BMI for all included patients was 22.8 (standard deviation 4.3) kg/m^2^. Among the eligible PIVCs, 455; 2,041; and 929 were categorized as underweight, normal weight, and overweight/obese, respectively. In the underweight group, catheter size ≥ 18 G and amiodarone administration were independently associated with the incidence of phlebitis. Drug administration standardization was associated with the reduction of phlebitis. In the normal weight group, elective surgery as a reason for ICU admission, and nicardipine, noradrenaline, and levetiracetam administration were independently associated with the incidence of phlebitis. Heparin administration was associated with the reduction of phlebitis. In the overweight/obese group, the Charlson comorbidity index, catheter size ≥ 18 G, and levetiracetam administration were independently associated with the incidence of phlebitis. Catheters made from PEU-Vialon (polyetherurethane without leachable additives) and tetrafluoroethylene were associated with the reduction of phlebitis.

**Conclusion:**

We investigated the risk factors for peripheral phlebitis according to BMI in ICU and observed different risk factors in groups stratified by BMI. For example, in underweight or overweight patients, large size PIVCs could be avoided. Focusing on the various risk factors for phlebitis according to patients’ BMIs may aid the prevention of phlebitis.

## Introduction

Peripheral intravascular catheters (PIVCs) are essential invasive devices for most patients in the intensive care unit (ICU) (1). Phlebitis is a common complication associated with PIVC use, occurring in 23.8% of catheterized patients and with 7.5% of inserted PIVCs in ICU (2, 3). Phlebitis can cause major problems, such as skin necrosis, infection (including infective endocarditis), pain, irritation, and treatment interruption (4–6).

Previous studies have identified risk factors for phlebitis in patients admitted to the ICU, including patient body mass index (BMI), ICU characteristics, the medical staff inserting a catheter, catheter insertion site, and medication type (7). In particular, the BMI of patients had a U-shaped relationship with the occurrence of phlebitis. The risk factors for phlebitis may differ between underweight and overweight patients. However, the specific risk of phlebitis in underweight, overweight, and normal weight patients remains unclear.

If the risk factors for phlebitis vary according to patients’ BMI, personalized prevention could be provided for phlebitis. Therefore, this study aimed to investigate the risk factors for phlebitis according to BMI in critically ill patients.

## Materials and methods

### Study design and setting

This study was a *post-hoc* analysis using the database of the Incidence And risk factors of phlebitis and coMplicatiOns due to peRipheral VENoUS catheters in critically ill patients (AMOR-VENUS) study. The AMOR-VENUS study was a prospective, multicenter, observational study conducted at 22 institutions and 23 ICUs in Japan between January and March 2018 (3, 7). The AMOR-VENUS study was registered at the University Hospital Medical Information Network Clinical Trials Registry (registration number: UMIN000028019) and approved by the institutional review board or medical ethics committee of each participating institution. The main objectives of the AMOR-VENUS study included an epidemiological investigation of the occurrence of phlebitis due to PIVC use in the ICU and an exploratory investigation of the risk factors for phlebitis. This study aimed to investigate the risk factors for PIVC-induced phlebitis according to BMI. The need for a new ethical review was waived for this study because the approval for the AMOR-VENUS study included the *post-hoc* analysis. This study was described according to the Strengthening the Reporting of Observational Studies in Epidemiology statement (8).

### Included patients and peripheral intravascular catheters

The AMOR-VENUS study database included consecutive patients aged ≥18 years who had PIVCs inserted during ICU admission. Details of the inclusion and exclusion criteria have been previously described in the AMOR-VENUS study article (3). In this *post-hoc* study, only PIVCs inserted after ICU admission were included to avoid immortal time bias. In addition, patients with missing BMI data were excluded from this study. Eligible patients were stratified into the following groups, based on their BMI: Underweight (BMI < 18.5 kg/m^2^), normal weight (18.5 ≤ BMI < 25.0 kg/m^2^), and overweight/obese (BMI ≥ 25.0 kg/m^2^) (9). Since there were only 73 obese patients, we analyzed overweight and obese patients together.

### Data collection

Data on the following patient characteristics were extracted: Age, sex, body height, body weight, BMI, Charlson comorbidity index, type of ICU admission, sepsis on ICU admission, mechanical ventilation, and acute physiology and chronic health evaluation (APACHE) II scores (10–12). Data on the following outcomes were also extracted: ICU and hospital mortality, length of ICU and hospital stay, and incidence of phlebitis. Data on participating ICU characteristics, including standardized drug administration measures in the ICU and education on venous catheter management for nurses, were collected. The standardized drug administration measures in the ICU in this study were defined according to documented standard operating procedures for drug administration supervised by a pharmacist at the relevant institution, which included the drug composition, choice of administration route, administration rate, and contraindications to compounding. In addition, data on the following PIVC characteristics were collected: Medical staff inserting the catheter (doctor, nurse, or medical technologist), insertion site, catheter materials, catheter gauge, T use, use of antiseptic solution before catheterization, use of ultrasonography, number of trials for insertion, difficulty with insertion, dressing use, infection during catheter dwell, and catheter dwelling duration. Information on the drugs (fentanyl, heparin, fat, nicardipine, dexmedetomidine, ampicillin/sulbactam, albumin, paracetamol, potassium, meropenem, ceftriaxone, steroids, vancomycin, magnesium, peripheral parenteral nutrition, phosphorus, carperitide, noradrenaline, midazolam, nitroglycerin, dobutamine, cefmetazole, amiodarone, cefepime, levetiracetam, and landiolol) administered using PIVCs during ICU stays was also extracted. Phlebitis was defined using the Phlebitis Scale developed by the Infusion Nurses Society (13). Details of the data collected in the original AMOR-VENUS study have been described previously (3).

### Outcome measures

In the main institution, a blinded assessor identified phlebitis and graded it into four grades based on six clinical symptoms (see Supplementary Tables 1,2) (13). Well-trained nurses assessed the severity of symptoms, including pain, in patients with disrupted consciousness, using facial, and behavioral pain scales. A pilot training program was established to diagnose phlebitis accurately and eliminate information bias. Moreover, during the study period, well-trained expert clinician-researchers at the central institution monitored diagnostic accuracy. The data management center confirmed the accuracy of the information on the catheter insertion locations using phlebitis photos submitted by each institution within the first month of data collection.

### Statistical analyses

The Shapiro–Wilk normality test and Kolmogorov–Smirnov normality test were used to test the normality of continuous variables of interest when the sample size was less than 2,000 and 2,000 or greater, respectively. Continuous variables are presented as mean and standard deviation for normal distributions, and median and interquartile range (IQR) for non-normal distributions. Categorical variables are presented as counts and percentages. Univariate analysis was performed using the one-way analysis of variance or Kruskal–Wallis test for continuous variables with the Bonferroni multiple comparison test as appropriate. The chi-square test and Fisher’s exact test were used for categorical variables as appropriate.

If a new catheter was inserted in the same patient in the ICU, we treated it as a separate catheter for analysis. Multivariate marginal Cox regression analysis was performed for each patient group (stratified by BMI) to explore potential risk factors associated with the incidence of phlebitis considering individual-level and institution-level clustering. The timing of PIVC insertion in the ICU was defined as the zero time point in the marginal Cox regression models. The occurrence of phlebitis, removal of the PIVC, or timing of ICU discharge (if the patient left the ICU with the PIVC *in situ*) was defined as censoring. All variables, including patients, participating ICUs, PIVCs characteristics, and administered drugs mentioned in the data collection section, except BMI, catheter dwelling duration, and outcomes, were used as explanatory variables in the analysis, as potential risk factors. Multivariate analysis was performed using the backward selection method, which was used for variable selection. In addition, a statistical interaction test was performed by entering an interaction term combining two of each of the selected variables in a multivariate model to examine effect modification. Effect estimates were described using hazard ratios (HR) and 95% confidence intervals (CIs).

All statistical tests were two-sided, and a *p*-value of < 0.05 was considered statistically significant. Statistical analyses were conducted using SAS Studio (SAS Institute Inc., Cary, NC, USA).

## Results

### Patient and peripheral intravascular catheter enrollment

**FIGURE 1:**
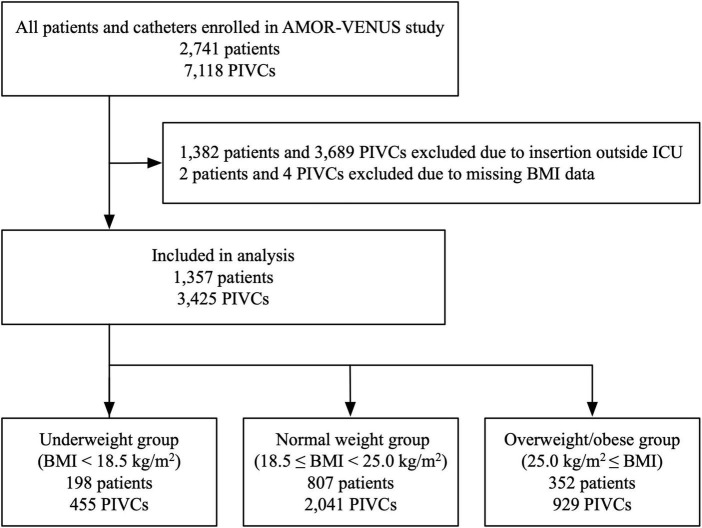
Flowchart of study participants. BMI, body mass index; ICU, intensive care unit; PIVC, peripheral intravascular catheter.

A total of 2,741 patients and 7,118 PIVCs from 23 ICUs were included in the AMOR-VENUS study database. Of these, 1,384 patients and 3,693 PIVCs were excluded. Finally, 1,357 patients and 3,425 PIVCs were analyzed (Figure 1).

### Grouping of eligible patients based on body mass index

The mean BMI for all included patients was 22.8 (standard deviation 4.3) kg/m^2^. Of the included patients and PIVCs, 198 (14.6%) patients and 455 (13.3%) PIVCs were categorized into the underweight group (BMI < 18.5 kg/m^2^); 807 (59.5%) patients and 2,041 (59.6%) PIVCs were categorized into the normal weight group (18.5 ≤ BMI < 25.0 kg/m^2^); and 352 (25.9%) patients and 929 (27.1%) PIVCs were categorized into the overweight/obese group (BMI ≥ 25.0 kg/m^2^) (Figure 1).

### Patients’ characteristics and outcomes

The characteristics of all the included patients and each BMI group are shown in Table 1. The missing data are described in Supplementary Table 3. Overall, the mean patient age was 69 (IQR, 59–77) years; 815 (60.1%) patients were men, 380 (28.0%) were medical patients, 341 (25.6%) required mechanical ventilation within 24 h of admission to the ICU, 53 (3.9%) died in the ICU, and 105 (7.7%) had phlebitis during the ICU stay. In addition, statistically significant differences in age, sex, Charlson comorbidity index, type of ICU admission, mechanical ventilation, APACHE II score, and length of hospital stay were observed in each BMI group. In multiple comparisons, the overweight/obese group was significantly younger than the other groups (vs. underweight group, *p* = 0.007; vs. normal weight group, *p* = 0.002, respectively). Charlson index in underweight group was higher than that in the overweight/obese group (*p* = 0.048). APACHE II score in underweight group was higher than that in the other groups (vs. normal group, *p* = 0.002; vs. overweight/obese group, *p* < 0.001, respectively). Length of hospital stay differed significantly among all three groups, with the underweight, normal weight, and overweight/obese group having the longest length of stay, in that order. The body height was not significantly different in each BMI group within mean differences of 2.9 cm.

**TABLE 1:** Patient demographics, characteristics, and outcomes on intensive care unit admission stratified by body mass index.

Variable	Overall (*n* = 1,357)	Underweight group (*n* = 198)	Normal weight group (*n* = 807)	Overweight/obese group (*n* = 352)	*p-value*
Age, median (IQR), years	69 (59–77)	70 (59–79)	70 (60–78)	68 (55–75)	0.001*
Male, *n* (%)	815 (60.1)	96 (48.5)	495 (61.3)	224 (63.6)	0.001^†^
Body height, mean (SD), cm	160.6 (9.9)	159.0 (9.0)	160.4 (9.9)	161.9 (10.2)	0.085^‡^
Body weight, mean (SD), kg	59.1 (14.2)	42.6 (6.2)	56.4 (8.3)	74.6 (13.8)	<0.001^‡^
BMI, mean (SD), kg/m^2^	22.8 (4.3)	16.8 (1.4)	21.8 (1.7)	28.3 (11.8)	<0.001^‡^
Charlson comorbidity index	4 (3–6)	4.50 (3–6)	4 (3–6)	4 (2–5)	0.026*
**Type of ICU admission, *n* (%)**					
Medical	380 (28.0)	67 (33.8)	222 (27.5)	91 (25.9)	0.024^†^
Emergency surgical	175 (12.9)	34 (17.2)	102 (12.6)	39 (11.1)	
Elective surgical	802 (59.1)	97 (49.0)	483 (59.9)	222 (63.1)	
**Sepsis on ICU admission, *n* (%)**					
Sepsis	48 (3.5)	10 (5.1)	29 (3.6)	9 (2.6)	0.16^†^
Septic shock	66 (4.9)	15 (7.6)	37 (4.6)	14 (4.0)	
Mechanical ventilation within 24 h of admission to ICU, *n* (%)	341 (25.6)	64 (33.0)	203 (25.6)	74 (21.6)	0.015^†^
APACHE II score, median (IQR)	13 (10–18)	15 (11–20)	13 (10–18)	12 (9–16)	<0.001*
Phlebitis, *n* (%)	105 (7.7)	21 (10.6)	58 (7.2)	26 (7.4)	0.26^†^
Length of ICU stay, median (IQR), days	1 (1–3)	1 (1–3)	1 (1–3)	1 (1–3)	0.13*
Length of hospital stay, median (IQR), days	20 (12–37)	26 (15–54)	20 (12–37)	17 (10–29)	<0.001*
ICU mortality, *n* (%)	53 (3.9)	8 (4.0)	30 (3.7)	15 (4.3)	0.90^†^
Hospital mortality, *n* (%)	108 (8.0)	22 (11.1)	59 (7.3)	27 (7.7)	0.20^†^

Continuous variables are presented as mean and standard deviation for normal distributions, and median and interquartile range for non-normal distributions. Categorical variables are presented as counts and percentages. APACHE, acute physiology and chronic health evaluation; BMI, body mass index; ICU, intensive care unit; IQR, interquartile ranges; SD, standard deviation. *Univariate analysis was performed using the Kruskal–Wallis test. ^†^Univariate analysis was performed using the chi-square test. ^‡^Univariate analysis was performed using the one-way analysis of variance.

### Peripheral intravascular catheter characteristics and outcomes

The characteristics of all the included PIVCs and each BMI group are shown in Table 2. The missing data are described in Supplementary Table 4. Overall, 3,377 PIVCs (98.6%) were inserted with the provision of standardized drug administration measures in the ICU; 2,391 (89.2%) were inserted by the nurse; 2,516 (74.1%) were inserted in the forearm; and 2,314 (68.8%) were sized ≤ 22 G. The median duration of catheter dwell was 46 h (IQR, 21–83 h). Phlebitis occurred in 313 (9.1%) PIVCs during the ICU stay and the median duration of catheter dwell until the incidence of phlebitis was 37 h (IQR, 19–58 h) in the patients with phlebitis. There were statistically significant differences in the insertion site and use of ultrasonography among the BMI groups. There were 674 (20.6%) missing data points for the variable on medical staff inserting the catheter, 48 (1.5%) for catheter gauge, and 9 (0.3%) for the duration of catheter dwell.

**TABLE 2:** Peripheral intravenous catheter characteristics during insertion stratified by body mass index.

Variable	Overall (*n* = 3,425)	Underweight group (*n* = 455)	Normal weight group (*n* = 2,041)	Overweight/obese group (*n* = 929)	*p-value*
Drug administration standardization, *n* (%)	3,377 (98.6)	453 (99.6)	2,011 (98.5)	913 (98.3)	0.15^†^
Education on venous catheter management for nurses, n (%)	1,977 (57.7)	248 (54.5)	1,212 (59.4)	517 (55.7)	0.053^†^
**Catheter inserted by: *n* (%)**					
Doctor	287 (10.7)	33 (9.5)	169 (10.6)	85 (11.5)	0.43^†^
Nurse	2,391 (89.2)	316 (90.5)	1,425 (89.4)	650 (88.3)	
Medical technologist	1 (0.0)	0 (0.0)	0 (0.0)	1 (0.1)	
**Insertion site, *n* (%)**					
Upper arm	356 (10.5)	61 (13.6)	218 (10.8)	77 (8.3)	0.006^†^
Forearm	2,516 (74.1)	341 (76.1)	1,484 (73.3)	691 (74.9)	
Elbow	163 (4.8)	13 (2.9)	100 (4.9)	50 (5.4)	
Lower limbs	360 (10.6)	33 (7.4)	222 (11.0)	105 (11.4)	
**Catheter material, *n* (%)**					
PEU-Vialon*	1,087 (31.7)	141 (31.0)	652 (31.9)	294 (31.6)	0.078^†^
Polyurethane	978 (28.6)	137 (30.1)	546 (26.8)	295 (31.8)	
Tetrafluoroethylene	1,288 (37.6)	170 (37.4)	800 (39.2)	318 (34.2)	
Others	72 (2.1)	7 (1.5)	43 (2.1)	22 (2.4)	
**Catheter gauge, *n* (%)**					
22 G or smaller size	162 (4.7)	11 (2.4)	113 (5.5)	38 (4.1)	0.075^†^
20 G	888 (26.4)	108 (24.2)	536 (26.7)	244 (26.8)	
18 G or larger size	2,314 (68.8)	328 (73.4)	1,359 (67.7)	627 (69.0)	
**Glove use, *n* (%)**					
None	115 (4.4)	15 (4.5)	63 (4.0)	37 (5.1)	0.82^‡^
Sterile	19 (0.7)	2 (0.6)	12 (0.8)	5 (0.7)	
Non-sterile	2,494 (94.9)	320 (95.0)	1,492 (95.2)	682 (94.2)	
**Antiseptic solution before catheterization, *n* (%)**					
None	8 (0.3)	3 (0.9)	3 (0.2)	2 (0.3)	0.25^‡^
Alcohol	2,597 (97.5)	339 (98.0)	1,543 (97.4)	715 (97.7)	
Chlorhexidine alcohol	53 (2.0)	4 (1.2)	36 (2.3)	13 (1.8)	
Povidone iodine	2 (0.1)	0 (0.0)	2 (0.1)	0 (0.0)	
Others	3 (0.1)	0 (0.0)	1 (0.1)	2 (0.3)	
Use of ultrasonography, *n* (%)	58 (2.2)	2 (0.6)	34 (2.2)	22 (3.0)	0.039^‡^
**Number of trials for insertion, *n* (%)**					
1	2,118 (80.9)	267 (79.7)	1,267 (81.4)	584 (80.4)	0.85^†^
2	312 (11.9)	44 (13.1)	176 (11.3)	92 (12.7)	
3	130 (5.0)	19 (5.7)	77 (4.9)	34 (4.7)	
≥4	57 (2.2)	5 (1.5)	36 (2.3)	16 (2.2)	
**Difficulty of insertion, *n* (%)**					
Easy	1,232 (47.5)	164 (49.4)	747 (48.3)	321 (44.9)	0.21^†^
Slightly easy	771 (29.7)	106 (31.9)	441 (28.5)	224 (31.3)	
Slightly difficult	455 (17.6)	45 (13.6)	283 (18.3)	127 (17.8)	
Difficult	134 (5.2)	17 (5.1)	74 (4.8)	43 (6.0)	
**Dressing, *n* (%)**					
Sterile	3,323 (98.0)	437 (97.3)	1,983 (98.1)	903 (98.0)	0.59^†^
Non-sterile	69 (2.0)	12 (2.7)	39 (1.9)	18 (2.0)	
Any infection during catheter dwell, *n* (%)	803 (23.4)	119 (26.2)	472 (23.1)	212 (22.8)	0.34^†^
Duration of catheter dwell, median (IQR), hours	46 (21–83)	45 (21–88)	46 (22–82)	47 (21–80)	0.95^§^
Phlebitis, *n* (%)	313 (9.1)	46 (10.1)	181 (8.9)	86 (9.3)	0.70^†^
Duration of catheter dwell until the incidence of phlebitis, median (IQR), hours	37 (19–58)	42 (18–61)	32 (19–56)	43 (20–67)	0.19^§^

Continuous variables are presented as mean and standard deviation for normal distributions, and median and interquartile range for non-normal distributions. Categorical variables are presented as counts and percentages. Continuous variables are presented as medians (interquartile ranges). Categorical variables are presented as counts (percentages). IQR, interquartile ranges, *PEU-Vialon: polyetherurethane without leachable additives. ^†^Univariate analysis was performed using the chi-square test. ^‡^Univariate analysis was performed using the Fisher’s exact test. ^§^Univariate analysis was performed using the Kruskal–Wallis test.

### Characteristics of the drugs administered

Table 3 shows the drug characteristics of the multivariate models. No data were missing. More than 300 drugs were administered to the patients in the AMOR-VENUS study, and 26 were administered to at least 5% of patients, with a phlebitis frequency of ≥1% (7). Fentanyl was the most administered drug (13.5%), followed by heparin (9.8%), fat emulsion (9.0%), and nicardipine (9.0%). In addition, statistically significant differences in nicardipine, meropenem, ceftriaxone, steroids, dobutamine, and cefepime were observed in each BMI group.

**TABLE 3:** Characteristics of the drugs administered with the catheter *in situ* stratified by body mass index.

Variable	Overall (*n* = 3,425)	Underweight group (*n* = 455)	Normal weight group (*n* = 2,041)	Overweight/obese group (*n* = 929)	*p-value*
Fentanyl, *n* (%)	462 (13.5)	57 (12.5)	273 (13.4)	132 (14.2)	0.67*
Heparin, *n* (%)	334 (9.8)	49 (10.8)	189 (9.3)	96 (10.3)	0.48*
Fat, *n* (%)	308 (9.0)	48 (10.5)	177 (8.7)	83 (8.9)	0.45*
Nicardipine, *n* (%)	307 (9.0)	29 (6.4)	176 (8.6)	102 (11.0)	0.013*
Dexmedetomidine, *n* (%)	292 (8.5)	31 (6.8)	166 (8.1)	95 (10.2)	0.062*
Ampicillin/sulbactam, *n* (%)	199 (5.8)	27 (5.9)	116 (5.7)	56 (6.0)	0.93*
Albumin, *n* (%)	176 (5.1)	30 (6.6)	108 (5.3)	38 (4.1)	0.12*
Paracetamol, *n* (%)	165 (4.8)	23 (5.1)	103 (5.0)	39 (4.2)	0.59*
Potassium, *n* (%)	154 (4.5)	21 (4.6)	80 (3.9)	53 (5.7)	0.093*
Meropenem, *n* (%)	135 (3.9)	13 (2.9)	70 (3.4)	52 (5.6)	0.008*
Ceftriaxone, *n* (%)	125 (3.6)	7 (1.5)	68 (3.3)	50 (5.4)	0.001*
Steroids, *n* (%)	125 (3.6)	24 (5.3)	56 (2.7)	45 (4.8)	0.003*
Vancomycin, *n* (%)	120 (3.5)	16 (3.5)	76 (3.7)	28 (3.0)	0.62*
Magnesium, *n* (%)	111 (3.2)	17 (3.7)	70 (3.4)	24 (2.6)	0.39*
PPN, *n* (%)	92 (2.7)	15 (3.3)	50 (2.4)	27 (2.9)	0.53*
Phosphorus, *n* (%)	91 (2.7)	13 (2.9)	59 (2.9)	19 (2.0)	0.40*
Carperitide, *n* (%)	88 (2.6)	11 (2.4)	56 (2.7)	21 (2.3)	0.73*
Noradrenaline, *n* (%)	87 (2.5)	15 (3.3)	52 (2.5)	20 (2.2)	0.45*
Midazolam, *n* (%)	60 (1.8)	2 (0.4)	41 (2.0)	17 (1.8)	0.068^†^
Nitroglycerin, *n* (%)	60 (1.8)	4 (0.9)	36 (1.8)	20 (2.2)	0.24^†^
Dobutamine, *n* (%)	50 (1.5)	0 (0.0)	36 (1.8)	14 (1.5)	0.018^†^
Cefmetazole, *n* (%)	48 (1.4)	7 (1.5)	31 (1.5)	10 (1.1)	0.61*
Amiodarone, *n* (%)	41 (1.2)	6 (1.3)	25 (1.2)	10 (1.1)	0.91*
Cefepime, *n* (%)	41 (1.2)	1 (0.2)	34 (1.7)	6 (0.6)	0.007^†^
Levetiracetam, *n* (%)	41 (1.2)	2 (0.4)	29 (1.4)	10 (1.1)	0.20^†^
Landiolol, *n* (%)	40 (1.2)	4 (0.9)	25 (1.2)	11 (1.2)	0.82^†^

Data are presented as counts (percentages). PPN, peripheral parenteral nutrition. *Univariate analysis was performed using the chi-square test. ^†^Univariate analysis was performed using the Fisher’s exact test.

### Risk factors for phlebitis in each body mass index group

Multivariate, multilevel, marginal Cox regression analyses were performed to determine the risk factors for phlebitis in each BMI group (Tables 4–6). In the underweight group, of the 303 analyzed PIVCs, phlebitis occurred in 36 (11.9%). Catheter size ≥ 18 G (≤22 G as reference; HR, 11.5; 95% CI, 2.48–53.3; *p* = 0.002) and amiodarone administration (HR, 12.34; 95% CI, 2.41–63.2; *p* = 0.003) were independently associated with the incidence of phlebitis. Drug administration standardization was associated with reduction in the incidence of phlebitis (HR, 0.01; 95% CI, 0.00–0.06; *p* < 0.001).

**TABLE 4:** Marginal Cox regression analysis for phlebitis in the underweight group.

Analyzed PIVCs (*n* = 303)		
Phlebitis incidence: 36/303 (11.9%)		
**Variables**	**HR (95% CI)**	***p-value***

**Catheter size**		
22 G or smaller size	Reference	
20 G	0.90 (0.37–2.19)	0.82
18 G or larger size	11.49 (2.48–53.25)	0.002
**Drug administration standardization**	0.01 (0.00–0.06)	< 0.001
**Amiodarone**	12.34 (2.41–63.18)	0.003

CI, confidence interval; HR, hazard ratio; PIVCs: peripheral intravenous catheters.

**TABLE 5:** Marginal Cox regression analysis for phlebitis in the normal weight group.

Analyzed PIVCs (*n* = 1,412)		
Phlebitis incidence: 137/1,412 (9.7%)		
**Variables**	**HR (95% CI)**	***p-value***

**Type of ICU admission**		
Medical	Reference	
Emergency surgical	1.70 (0.69–4.22)	0.25
Elective surgical	2.55 (1.12–5.84)	0.027
**Nicardipine**	2.18 (1.38–3.43)	< 0.001
**Noradrenaline**	2.48 (1.30–4.73)	0.006
**Levetiracetam**	2.73 (1.20–6.21)	0.017
**Heparin**	0.36 (0.16–0.82)	0.016

CI, confidence interval; HR, hazard ratio; ICU, intensive care unit; PIVCs: peripheral intravenous catheters.

**TABLE 6:** Marginal Cox regression analysis for phlebitis in the overweight/obese group.

Analyzed PIVCs (*n* = 648)		
Phlebitis incidence: 66/648 (10.2%)		
**Variables**	**HR (95% CI)**	***p-value***

**Charlson comorbidity index**	1.14 (1.04–1.24)	0.004
**Catheter material**		
Polyurethane	Reference	
PEU-Vialon*	0.33 (0.17–0.64)	0.001
Tetrafluoroethylene	0.49 (0.27–0.89)	0.019
**Catheter size**		
22 G or smaller size	Reference	
20 G	0.57 (0.27–1.19)	0.13
18 G or larger size	26.81 (3.41–211.00)	0.002
**Levetiracetam**	12.95 (3.75–44.70)	< 0.001

*PEU-Vialon, polyetherurethane without leachable additives. CI, confidence interval; HR, hazard ratio; PIVCs, peripheral intravenous catheters.

In the normal weight group, of the 1,412 analyzed PIVCs, phlebitis occurred in 137 (9.7%). Elective surgery as a reason for ICU admission (medical as reference; HR, 2.55; 95% CI, 1.12–5.84; *p* = 0.027), and nicardipine (HR, 2.18; 95% CI, 1.38–3.43; *p* < 0.001), noradrenaline (HR, 2.48; 95% CI, 1.30–4.73; *p* = 0.006), and levetiracetam (HR, 2.73; 95% CI, 1.20–6.21; *p* = 0.017) administration were independently associated with the incidence of phlebitis. Heparin administration was independently associated with reduction in the incidence of phlebitis (HR, 0.36; 95% CI, 0.16–0.82; *p* = 0.016).

In the overweight/obese group, of the 648 analyzed PIVCs, phlebitis occurred in 66 (10.2%). The Charlson comorbidity index (HR, 1.14; 95% CI, 1.04–1.24; *p* = 0.004), catheter size ≥ 18 G (≤22 G as reference; HR, 26.8; 95% CI, 3.41–211.0; *p* = 0.002), and levetiracetam (HR, 13.0; 95% CI, 3.75–44.7; *p* < 0.001) administration were independently associated with the incidence of phlebitis. Polyetherurethane without leachable additives (PEU-Vialon) (polyurethane as reference; HR, 0.33; 95% CI, 0.17–0.64; *p* = 0.001) and tetrafluoroethylene (polyurethane as reference; HR, 0.49; 95% CI, 0.27–0.89; *p* = 0.019) were independently associated with reduction in the incidence of phlebitis.

The results of the interaction analysis showed that no combination of variables was a significant interaction term in each BMI group.

## Discussion

### Main findings

We aimed to evaluate the risk factors for phlebitis according to BMI in critically ill patients. Eligible patients were stratified into three groups based on their BMI (9). In each BMI group, varying risk factors for phlebitis incidence were identified after multilevel marginal Cox regression analysis. In the underweight group, catheter size and amiodarone administration were independently associated with the incidence of phlebitis. Further, drug administration standardization was associated with the reduction of phlebitis. In the normal weight group, elective surgery as a reason for ICU admission and nicardipine, noradrenaline, and levetiracetam administration were independently associated with the incidence of phlebitis. Heparin administration was associated with the reduction of phlebitis. In the overweight/obese group, the Charlson comorbidity index, catheter size, and levetiracetam administration were independently associated with the incidence of phlebitis. Catheters made from PEU-Vialon and tetrafluoroethylene were associated with the reduction of phlebitis.

Standardized drug administration measures in the ICU might have reduced the risk for phlebitis in the underweight group. The standard method of drug administration in the ICU in this study was defined in detail according to the documented standard operating procedures for drug administration. The Society of Critical Care Medicine recommended that ICU pharmacists monitor the appropriateness of drug administration, including the regimen and potential for drug interactions, but did not indicate specific methods (14). A method of administering drugs with a high-risk of causing phlebitis according to predetermined rules may reduce the risk of phlebitis, especially in frail patients, such as those who are underweight.

In this study, large-gauge PIVCs were associated with phlebitis in patients who were underweight and those who were overweight/obese. Larger-gauge PIVCs can obstruct the lumen of vessels, increasing the risk of thrombophlebitis and patient discomfort (15, 16). A *post-hoc* analysis of randomized controlled trial in three acute care hospitals showed that a larger diameter PIVC was significantly associated with phlebitis (HR, 1.48; 95% CI, 1.08–2.03) (16). The Infusion Therapy Standards of Practice published by the Infusion Nurse Society recommends the selection of the smallest-gauge PIVC that will accommodate the prescribed therapy (17). PIVC size may have a significant influence on phlebitis in underweight or overweight/obese patients.

In overweight/obese patients, the catheter material was associated with phlebitis. The insertion angle may vary in obese patients, and the material and stiffness of the catheter may be more likely to irritate blood vessels (18). In an observational study examining the insertion angle of PIVCs using ultrasonography, PIVCs inserted at a lower angle were associated with a lower incidence of phlebitis (18).

The Charlson comorbidity index score in the overweight/obese group, and the type of ICU admission in the normal weight group, were associated with phlebitis. These associations reflect the underlying background of the patients. A retrospective study conducted in general ward has reported that comorbidity was an independent risk factor for phlebitis (odds ratio, 1.48; 95% CI, 1.17–1.86), which is consistent with the results of previous studies (19).

Many drugs are administered to critically ill patients admitted into the ICU. Various studies have reported on the effect of administered drugs (including nicardipine, noradrenaline, and amiodarone) on the incidence of phlebitis (20–24). The results of this study also showed that these drugs were associated with the occurrence of phlebitis. High-risk drugs for PIVC-related complications, such as nicardipine and noradrenaline, should be administered using a central venous catheter. However, there are concerns regarding complications, such as bloodstream infection and bleeding, with the use of central venous catheters (25). The best catheter for administering high-risk drugs remains unclear. Therefore, selecting a device for high-risk drug administration according to BMI may be reasonable. In this study, levetiracetam was associated with phlebitis in all the BMI categories. Levetiracetam may cause venous irritation because of its high osmolality value in reconstitution or undiluted presentation (26). We may need to be cautious about how levetiracetam is dissolved.

### Clinical applications and strengths of the present study

To our knowledge, no study has examined the risk factors for phlebitis according to BMI. Various factors contribute to the incidence of PIVC-induced phlebitis in critically ill patients. Body size is relatively easy for healthcare providers to assess visually. If the risk factors for developing phlebitis are known for each body size, close follow-up according to the risk factors will enable early detection and treatment of phlebitis.

### Study limitations

This study has several limitations. First, we could not examine the risk factors for phlebitis based on its severity and only examined the risk factors for all types of phlebitis, including low-grade phlebitis. The severity of phlebitis included in the AMOR-VENUS study was mostly Grade 1 (73.8%). Grade 3 and 4 phlebitis accounted for only 4.5% of cases (7). Therefore, it is unclear whether the results of this study can be applied to more severe cases of phlebitis. Second, only clinically important factors and drugs associated with the incidence of phlebitis were included in the multivariate analyses in this study. However, it is impossible to determine a causal relationship between each factor and the incidence of phlebitis. In addition, there may have been unmeasured risk factors. Third, we performed Cox regression analysis with drug administration as a binary variable; we could not evaluate the duration and rate of drug administration and the dose. Fourth, the AMOR-VENUS study was conducted in Japan, and the average BMI of Japanese individuals is lower than that of individuals in the United States and Europe (27). Therefore, the external validity of the results of this study may not have been maintained. Finally, the reduction in the number of events owning to stratification by BMI may destabilize the robustness of the model, although we used the backward method of variable selection. Owning to differences in sample size and number of events when stratified by BMI, the probability of detecting significance may differ even when the same model is analyzed.

## Conclusion

Various factors contribute to the incidence of PIVC-induced phlebitis in critically ill patients. We found that the risk factors for phlebitis varied according to BMI. For example, in underweight or overweight patients, large size PIVCs could be avoided. In addition, follow-up while considering these different risk factors may prevent the occurrence of phlebitis. Careful monitoring may be necessary when using certain drugs, such as nicardipine, noradrenaline, and levetiracetam.

## Data availability statement

The datasets presented in this article are not readily available due to *post hoc* analyses by the co-authors. Requests to access the datasets should be directed to the corresponding author, HY (yasudahideto@me.com).

## Ethics statement

This study on human participants because the AMOR-VENUS study was registered at the University Hospital Medical Information Network Clinical Trials Registry (registration number: UMIN000028019) and approved by the Institutional Review Board or Medical Ethics Committee of each participating institution. The main objectives of the AMOR-VENUS study included an epidemiological investigation of the occurrence of phlebitis due to PIVC use in the ICU and an exploratory investigation of the risk factors for phlebitis. This study aimed to investigate the risk factors for PIVC-induced phlebitis according to BMI. The need for a new ethical review was waived for this study because the approval for the AMOR-VENUS study included *post-hoc* analysis. Written informed consent for participation was not required for this study in accordance with the national legislation and the institutional requirements.

## Author contributions

MK and HY conceptualized this study. MK performed the analyses, interpreted the data, and drafted the manuscript. HY supervised the statistical analysis. All authors contributed substantially to the study design, approved the manuscript, and agreed to be accountable for this work.

## Abbreviations

AMOR-VENUS: Incidence And risk factors of phlebitis and coMplicatiOns due to peRipheral VENoUS catheter in critically ill patients
APACHE: acute physiology and chronic health evaluation
BMI: body mass index
CI: confidence interval
HR: hazard ratio
ICU: intensive care unit
IQR: interquartile range
PEU-Vialon: polyetherurethane without leachable additives
PIVC: peripheral intravascular catheter.
