# Supplementary material for: Risk factors for peripheral venous catheter-related phlebitis stratified by body mass index in critically ill patients: A post-hoc analysis of the AMOR-VENUS study
Source: Front Med (Lausanne). 2022 Nov 28;9:1037274. doi: 10.3389/fmed.2022.1037274 (PMC9742461; doi:10.3389/fmed.2022.1037274)
Supplement: Supplementary file 1 [file Data_Sheet_1.docx]

**Additional file 1**

**Supplementary Table 1.** Definition of phlebitis according to the Infusion Nurses Society

| Grade | Definition |
| --- | --- |
| 0 | No clinical signs. |
| 1 | Erythema at the puncture site regardless of the presence or absence of pain. |
| 2 | Pain at the puncture site with erythema and/or edema. |
| 3 | Pain at the puncture site with erythema and/or edema and streak formation or a palpable venous cord. |
| 4 | Pain at the puncture site with erythema and/or edema and streak formation or a palpable venous cord > 1 inch. |
|  | Presence of purulent drainage. |

**Supplementary Table 2.** Definition of each variable for phlebitis according to the Infusion Nurses Society

| Variable | Definition |
| --- | --- |
| Pain | Pain around the catheter insertion site. |
| Erythema | Erythema around the catheter insertion site. |
| Edema | Swelling around the catheter insertion site. |
| Streak formation | Erythema along the blood vessels from the catheter insertion site. |
| Palpable venous cord | Induration along the blood vessels from the catheter insertion site. |
