# Supplementary material for: Risk factors for peripheral venous catheter-related phlebitis stratified by body mass index in critically ill patients: A post-hoc analysis of the AMOR-VENUS study
Source: Front Med (Lausanne). 2022 Nov 28;9:1037274. doi: 10.3389/fmed.2022.1037274 (PMC9742461; doi:10.3389/fmed.2022.1037274)
Supplement: Supplementary file 2 [file Data_Sheet_2.docx]

**Additional file 2**

**Supplementary Table 3.** Number of analyzed patients with missing data

|  | Overall  (n = 1357) | Underweight group  (n = 198) | Normal weight group  (n = 807) | Overweight/obese group  (n = 352) |
| --- | --- | --- | --- | --- |
| Age | 0 (0.0) | 0 (0.0) | 0 (0.0) | 0 (0.0) |
| Male | 0 (0.0) | 0 (0.0) | 0 (0.0) | 0 (0.0) |
| Body height | 0 (0.0) | 0 (0.0) | 0 (0.0) | 0 (0.0) |
| Body weight | 0 (0.0) | 0 (0.0) | 0 (0.0) | 0 (0.0) |
| BMI | 0 (0.0) | 0 (0.0) | 0 (0.0) | 0 (0.0) |
| Charlson comorbidity index | 0 (0.0) | 0 (0.0) | 0 (0.0) | 0 (0.0) |
| Type of ICU admission | 0 (0.0) | 0 (0.0) | 0 (0.0) | 0 (0.0) |
| Sepsis at ICU admission | 0 (0.0) | 0 (0.0) | 0 (0.0) | 0 (0.0) |
| Mechanical ventilation within 24 h of admission to ICU | 27 (2.0) | 4 (2.0) | 13 (1.6) | 10 (2.8) |
| APACHE II score | 107 (7.9) | 11 (5.6) | 65 (8.1) | 31 (8.8) |
| Phlebitis | 0 (0.0) | 0 (0.0) | 0 (0.0) | 0 (0.0) |
| Length of ICU stay | 0 (0.0) | 0 (0.0) | 0 (0.0) | 0 (0.0) |
| Length of hospital stay | 0 (0.0) | 0 (0.0) | 0 (0.0) | 0 (0.0) |
| ICU mortality | 0 (0.0) | 0 (0.0) | 0 (0.0) | 0 (0.0) |
| Hospital mortality | 0 (0.0) | 0 (0.0) | 0 (0.0) | 0 (0.0) |

Data are presented as counts (percentages) for all variables.

APACHE, acute physiology and chronic health evaluation; BMI, body mass index; ICU, intensive care unit

**Supplementary Table 4.** Number of analyzed peripheral intravenous catheters with missing data

|  | Overall  (n = 3425) | Underweight group  (n = 455) | Normal weight group  (n = 2041) | Overweight/obese group  (n = 929) |
| --- | --- | --- | --- | --- |
| Drug administration standardization | 0 (0.0) | 0 (0.0) | 0 (0.0) | 0 (0.0) |
| Education on venous catheter management for nurses | 0 (0.0) | 0 (0.0) | 0 (0.0) | 0 (0.0) |
| Catheter inserted by | 746 (21.8) | 106 (23.3) | 447 (21.9) | 193 (20.8) |
| Insertion Site | 30 (0.9) | 7 (1.5) | 17 (0.8) | 6 (0.6) |
| Catheter material | 0 (0.0) | 0 (0.0) | 0 (0.0) | 0 (0.0) |
| Catheter gauge | 61 (1.8) | 8 (1.8) | 33 (1.6) | 20 (2.2) |
| Glove use | 797 (23.3) | 118 (25.9) | 474 (23.2) | 205 (22.1) |
| Antiseptic solution before catheterization | 762 (22.2) | 109 (24.0) | 456 (22.3) | 197 (21.2) |
| Use of ultrasonography | 791 (23.1) | 115 (25.3) | 470 (23.0) | 206 (22.2) |
| Number of trials for insertion | 808 (23.6) | 120 (26.4) | 485 (23.8) | 203 (21.9) |
| Difficulties with insertion | 833 (24.3) | 123 (27.0) | 496 (24.3) | 214 (23.0) |
| Dressing | 33 (1.0) | 6 (1.3) | 19 (0.9) | 8 (0.9) |
| Any infection during catheter dwell | 0 (0.0) | 0 (0.0) | 0 (0.0) | 0 (0.0) |
| Duration of catheter dwell | 13 (0.4) | 2 (0.4) | 9 (0.4) | 2 (0.2) |
| Phlebitis | 0 (0.0) | 0 (0.0) | 0 (0.0) | 0 (0.0) |
| Time from catheter insertion to phlebitis | 0 (0.0) | 0 (0.0) | 0 (0.0) | 0 (0.0) |

Data are presented as counts (percentages) for all variables.
